# Editorial: Multidrug gram-negative bacilli : current situation and future perspective

**DOI:** 10.3389/fcimb.2023.1327413

**Published:** 2023-11-09

**Authors:** Luis Esaú López-Jácome, Rafael Franco-Cendejas, Rodolfo García-Contreras, Elvira Garza-González

**Affiliations:** ^1^ Laboratorio de Infectología, Instituto Nacional de Rehabilitación, Ciudad de México, Mexico; ^2^ Departamento de Microbiología y Parasitología, Facultad de Medicina, Universidad Nacional Autónoma de México (UNAM), Mexico City, Mexico; ^3^ Laboratorio de Microbiología Molecular, Facultad de Medicina, Universidad Autónoma de Nuevo León, Monterrey, Mexico

**Keywords:** drug resistance, multidrug-resistant, carbapenems, acinetobacter, carbapenemase

Drug resistance in Gram-negative is increasing worldwide, complicating therapies of infections, and is associated with increased morbidity and mortality (Antimicrobial Resistance Collaborators, 2023). The World Health Organization designed and proposed a unified One Health approach for the study of resistance to antibiotics. This concept involves the interaction of humans and animals in the environment (Accessed on October 23, 2023).

In this topic we were dedicated to the study of multidrug-resistant Gram negatives and nine original articles from 69 authors were included. Among Gram-negative bacilli with multi-drug resistance, 3 studies included *Acinetobacter* and other studies included other relevant genera (*Salmonella, Proteus*, *Klebsiella*, and *Campylobacter*). Among the nine studies, one was a clinical study, specifically in patients with acute pancreatitis, two analyzed the colonization by drug-resistant organisms in humans and chickens and 6 other studies analyzed the molecular characteristics of particular strains. In this scenario, we included the study of multidrug-resistant bacteria in humans and animals with which we approach the One Health concept.

In the clinical study, Yan et al. established a model for early prediction of the risk of death in patients with acute pancreatitis infected with Gram negatives. In the results of this study, four variables were selected, but only two of them had an adequate confidence interval, with the carbapenem resistance showing the highest odds ratio value (OR 7.99), followed by the presence of septic shock (OR 6.33). This result underlined the importance of drug resistance in the outcome of patients, specially for broad-spectrum antibiotics such as carbapenems.

Also, two studies of carriers of drug-resistance genes were included, with one of them analyzing carriers in humans and the other in chickens. The first carriers study determined the relationship between the intestinal loads of *bla*_CTX-M-1_, *bla*_OXA-1_, *bla*_OXA-48,_ and *bla*_VIM_ genes and antibiotic consumption among 90 pediatric critically ill patients (Dahdouh et al.). In this study, 74.45% of patients were positive for at least one of the tested genes. Also, consumption of carbapenems, non-carbapenem β-lactams, and glycopeptides was associated with a negative result for *bla*_CTX-M-1_ and *bla*_OXA-1_ and the consumption of trimethoprim/sulfamethoxazole and aminoglycosides was associated with a negative result for bla_OXA-48._ The high prevalence of intestinal carriers of some carbapenemase encoding genes (*bla*_OXA-48_ and *bla*_VIM)_ puts the spotlight on the high possibility of dissemination of these genes because being in the intestinal lumen is an easy way to disseminate them to the hospital environment, healthcare providers, and other patients. The second one focused on the description of whole genome sequencing and determination of susceptibility patterns of 13 *Campylobacter jejuni* and 17 *Campylobacter coli* strains isolated from chickens in Chin**a**. This work was conducted by Xiao et al.. The results showed two dominant clonal complexes in *C. coli* (CC-354 *C. jejuni* and CC-828). All strains were resistant to ciprofloxacin and tetracycline and this phenotype correlated strongly with the presence of the GyrA T86I and *tet(O)*/*tet(L)* mutation, respectively. The high distribution of genes encoding resistance to quinolones and tetracycline in animals of high consumption in humans allows us to understand the relevance of studying animals in conjunction with humans, to, after knowing the magnitude of the problem, design strategies for its control.

Six articles were related to the microbiological and molecular analysis of clinical isolates and is not surprising that 3 of them were related to *Acinetobacter* species, one to *Salmonella enterica*, one for *Proteus mirabilis*, and one to *Klebsiella pneumoniae.*

In the first study Sharma et al., described the susceptibility profile of carbapenem-resistant *A. baumannii* co-harboring *bla*_OXA-23_ and metallo-β-lactamases against standard drugs and some combinations of drugs. They included 356 clinical isolates, with 89.04% being resistant to imipenem, 79.49% to meropenem, 77.80% to doripenem, 71.62% to ampicillin/sulbactam and 2.52% to colistin. The majority (87.69%) were co-producers of classes D and B carbapenemases. Regarding the drug combinations, there was synergy with meropenem-sulbactam (47%) and meropenem-colistin (57%), but reduced synergy was detected for those strains harboring the *bla*_NDM_ gene. The presence of the *bla*_NDM_ gene was a significant cause of synergy loss in meropenem-sulbactam and meropenem-colistin, further reducing therapeutic options for infections due to bacteria that had genes encoding the NDM gene. The presence of the NDM gene, beyond representing resistance to carbapenems and other antibiotics, may hinder the therapeutic efficacy of antibiotic combinations.

*Acinetobacter baumannii* is one of the species that have shown the worldwide distribution of multi- and extensive drug resistance. For this bacterial species, colistin is one of the few therapeutic alternatives. For the study of colistin resistance in *A. baumannii*, it is common the analysis of colistin-resistant strains, but the study of susceptible isolates is relevant to discriminating which mutations may be associated with resistance and which not. Zafer et al. analyzed 18 multi-/extensively drug-resistant *A. baumannii* isolates by whole genome sequencing with 17 of them being susceptible to colistin. All these strains carried missense mutations in *pmrCAB* and *lpxACD* operons. Overall, 34 mutations were found, 20 strains had substitutions in *pmrC* and no mutations were found in *pmrA* or *lpxA*. This study provides information that may be helpful in the study of colistin resistance mechanisms.

The third work about *Acinetobacter* genus describes the genetic characterization of carbapenem-resistant *Acinetobacter johnsonii*, co-producing NDM-1, OXA-58, and PER-1 collected for sputum. Surprisingly, the strain carried 11 plasmids, with *bla*_OXA-58_ and *bla*_PER-1_ genes located in the pAYTCM-1 plasmid that has been reported in several countries (Tian et al.). The *bla*_NDM-_ gene was located in conjugative plasmids that were stable even after 70 passages under antibiotics-free conditions.

Colistin resistance has been associated with a chromosomal mutation in genes associated with the modification of the lipid A of lipopolysaccharide, the primary target of colistin (DOI: 10.3389/fmicb.2014.00643). A plasmid-mediated colistin resistance was reported in 2015 (DOI: 10.1016/S1473-3099(15)00424-7), and this plasmid has been reported worldwide. Sun et al. reported the characteristics of 12 *mcr*-bearing plasmids in clinical *Salmonella enterica* in China (10 carried the *mcr*-1 and two carried the *mcr*-3). They detected that the *mcr* gene in clinical *Salmonella* was commonly carried by broad-host plasmids and had the potential to transfer into other bacteria by these plasmids.

The last study included the genetic analysis of resistance and virulence characteristics of clinical multidrug-resistant *Proteus mirabilis* isolates and detected 14 MDR bacteria that were susceptible to carbapenems (except imipenem), ceftazidime, and amikacin; as well as most of them were susceptible to aminoglycosides (Li et al.). Genomic analysis showed high genetic diversity, with integrative and conjugative elements commonly detected, carrying abundant antimicrobial resistance genes, including the *bla*_CTX-M-65_. The findings highlight the important roles of antimicrobial resistance genes in mediating the spread of antimicrobial resistance genes in *P. mirabilis* strains.

Finally, the last one detailed the whole bacterial genome of *K. pneumoniae* strain F4 resistant to routinely used antibiotics, including tigecycline associated with the presence of the *oqxAB* gene localized on the F4_chromosome and tmexCD1-toprJ1 on F4_plasmid A (Qu et al.).

This study showed a wide antibiotic resistance of *K. pneumoniae* strain F4 that effective antibiotics were virtually unavailable, therefore their spread and prevalence should be strictly controlled. Together, all these 9 studies contribute to better knowledge about the current situation and perspectives on infections by multidrug-resistant Gram-negative bacilli. Continued education on this topic may allow us to better understand the dynamics of transmission of these infections, and the role of the different participants to better implement control therapies.

## Author contributions

LL-J: Methodology, Writing – review & editing. RF-C: Writing – review & editing. RG-C: Writing – review & editing. EG-G: Methodology, Writing – original draft, Writing – review & editing.
